# International Dental Federation: Meetings of Executive Council, Subcommittee, and International Commission of Education

**Published:** 1902-01

**Authors:** 


### INTERNATIONAL DENTAL FEDERATION: MEETINGS OF EXECUTIVE COUNCIL, SUBCOMMITTEE, AND INTERNATIONAL COMMISSION OF EDUCATION.

### EXECUTIVE COUNCIL.

*Paris, August 15, 1901.*

The first meeting of the Executive Council of the International Dental Federation convened in Paris August 15, 1900, in the Ecole Dentaire.

The meeting was called to order at 10.30 A.M., the following members being present: Aguilar, of Madrid; Cunningham, of Cambridge; Förberg, of Stockholm; Godon, of Paris; Harlan, of Chicago; Sauvez, of Paris.

Dr. Godon, as president of the Congress, presided at the meeting, and invited the assembly to elect its members.

It was decided that the Executive Council should have a president, two vice-presidents, and a secretary-treasurer.

Dr. Godon was unanimously elected president; Drs. Harlan and Hesse vice-presidents, and Dr. Sauvez secretary-treasurer.

Dr. Godon thanked the assembly for the honor extended to him; and took the chair.

It was decided that the discussions at this meeting should be in French; also that the official communications should be issued in the French language.

The title adopted by the General Assembly, August 14, 1900, "Conseil Executif de la Fédèration Dentaire Internationale," was adopted, this title to be always in French.

It was agreed upon that the membership of the Council should be as follows:

The Executive Council of the International Dental Federation shall be composed of the nine members elected by the Third International Dental Congress, and of such adjunct members as shall be elected by the Executive Council after consultation with the national committees.

The following decisions were then adopted:

That the Council represents the dental profession without distinction of nationality.

That the present officers shall hold their appointments until the next meeting of the Council, which will be held in England, in August, 1901.

That the powers of the Council shall expire at the next International Dental Congress.

That vacancies shall be filled by the Council.

That the expenses shall be equally divided among the members.

That the duties of the Council are:

*First.* To prepare a set of by-laws for the International Dental Federation, to be adopted at its next meeting.

*Second.* To decide upon the place of meeting and date of the next Congress.

*Third.* To appoint the International Commission of Education and the other commissions that it may seem advisable to appoint.

A Committee of three was then appointed for the purpose of preparing the by-laws and regulations for the International Dental Federation. Drs. Godon, Sauvez, and Cunningham were elected to constitute this committee. In order to give validity to the decisions of the Council it was decided that the consent of at least three members shall be necessary.

The Council then proceeded to the election of the International Commission of Education.

The following members were appointed: Drs. Kirk, Cunningham, Sandstedt, Queudot, Brophy, Hesse, Aguilar, Martinier, Patterson, Arkövy, Godon, Guillermin, Grevers, Giaria, Limberg, Rosenthal, and Burne.

The officers of the Council are *ex-officio* members of all the commissions, and preside over their meetings until the time of the election of their own officers.

A report upon the organization of the Commission of Education shall be prepared by the executive officers and presented at the next meeting in England.

Adjourned.

EXECUTIVE COUNCIL (SUBCOMMITTEE).

*Paris, November 28, 1900.*

The meeting was called to order by Dr. Godon.

The Subcommittee acted favorably upon the following resolutions:

That an international review to be known as the *Bulletin of the Executive Council* shall be issued; that the first number of this review shall appear December 30, 1900, and that it should be printed in French, English, and Spanish, in *l'Odontologie;* also that four separate reprints of said bulletin shall be sent to the members of the Council.

That the members of the Executive Council shall be notified of their appointment by a letter signed by the president and secretary-general.

That the resolutions adopted at the closing session of the Congress shall be sent to the presidents and secretaries of the national committees, asking them to carry out these resolutions; also those voted by the Executive Council at its meeting of August 15, 1900.

That the members of the Commission of Education shall be notified of their appointment to said committee.

That in the first number of the bulletin the list of the members of the Commission of Education shall appear.

It was agreed that Drs. Godon and Sauvez should prepare the by-laws, and that they should submit the result of their work to Dr. Cunningham.

Adjourned.

*Paris, May 27, 1901.*

The meeting was called to order at 8.30 P.M., with Dr. Godon in the chair.

The following resolutions were adopted at this meeting:

1. That the International Dental Federation shall meet in London at the time of the meeting of the British Dental Association.

2. That the Executive Council shall examine at its first meeting in London, on Sunday, August 4, the proposed regulations for the International Dental Federation which the subcommittee has been charged to prepare.

3. That the International Commission of Education shall also meet on the same day in London.

4. That this committee shall meet for the second time in Cambridge, August 7, after the meeting of the British Dental Association.

5. That a second meeting of the Executive Council shall be held after the meeting of the International Commission of Education; this session to be the closing one.

6. That a general meeting of the Executive Council of the International Commission of Education shall take place in Cambridge, August 1, 1901, and that to this meeting delegates of the national committees, societies, or members of the national societies or federations who took part in the organization of the Congress of 1900, as well as the special guests of the Executive Council, shall be invited.

7. That the last meetings of the council shall be held in the buildings of the University of Cambridge.

8. That a banquet shall close this first session of the International Dental Federation.

9. That the power of appointing delegates shall rest with the Executive Council or with the national societies and federations.

10. That announcements concerning these meetings, and a report of the proceedings of the sessions of the subcommittee, and all necessary information, shall be sent out to interested parties.

Adjourned at 11.30.

*London, August 3, 1901.*

The meeting of the Subcommittee was held in the Royal Temple Yacht Club.

The minutes of the last session were read and approved.

On motion of Dr. Godon, it was decided to submit to the Executive Council, at its meeting of August 4, the following decisions that had been adopted by the Subcommittee:

That the Executive Council shall proceed to election of officers for the year 1901-1902, and in particular to the election of a president. Confirmation is given the appointment of Dr. Frank, president of the Society of Austrian *Médecins Dentistes,* as adjunct member of the Executive Council for the meetings of the International Dental Federation of 1901, in place of Dr. Pichler.

That the following foreign delegates now in London shall be appointed adjunct members of the Commission of Education for the session of 1901. The subcommittee requests in particular the following appointments: Dr. Haderup, delegate of the Society of Dentists of Denmark; Drs. Baruch, Quartermann, and Thiet, delegates of the Association of Dentists of Belgium; Dr. Viau, delegate of the Society of the Ecole Dentaire of Paris; Dr. Choquet, delegate of the Société d'Odontologie of Paris; Drs. Frank, Weiser, and Zsigmondy, delegates of the Societies of Physicians of Austria. Any of these members may be appointed by the Executive Council to the Commission of Education.

The members of the Executive Council are *ex-officio* members of the Commission of Education.

The report of the secretary-general was adopted.

It was decided that the next meeting of the Executive Council should be held August 5, at four P.M., and that the Commission of Education should be composed of a president, two vice-presidents, and a secretary.

Adjourned at seven P.M.

EXECUTIVE COUNCIL.

*London, August 4, 1901.*

The meeting was called to order at 9.10 A.M. by President Godon.

The following members were present: Drs. Aguilar, Cunningham, Förberg, Harlan, Hesse, and Sauvez. The following were absent: Drs. Grevers and Pichler.

Before acting upon the regular order of business, the secretary-general read a letter from Dr. Pichler, of Vienna, in which he presented his resignation, and announced that the Society of *Médecins Dentistes* of that city has appointed Dr. Frank president of the society, and Drs. Zsigmondy and Weiser delegates of the society to the London meeting.

He then said that the committee proposed that Dr. Pichler's resignation should be accepted, as he had been obliged to take this step on account of illness, and that a letter be sent him expressing the regrets of the Council; also that Dr. Frank be installed member of the Council for the present session.

This recommendation was approved by the Council.

The president then said that before beginning with the order of business it would be advisable to determine the hour and date of the next meeting.

The Council designated August 5, at four P.M.

The secretary-general then read the minutes of the meeting of August 15, 1900.

Dr. Aguilar remarked that the *rôle* of the Subcommittee had been limited to the preparation of the by-laws.

The minutes were then approved.

The secretary-general then read the minutes of the meetings of the Subcommittee held in Paris, November 28, 1900, and May 27, 1901, and in London, August 3, 1901.

After the reading of these minutes Dr. Aguilar said that the Subcommittee had exceeded its powers when it occupied itself with questions other than the preparation of the by-laws, and asked that the minutes should be considered not as the proceedings of the Subcommittee, but as those of the Council.

Drs. Hesse and Frank objected to this, saying that it did not make any difference which body had been instrumental in the preparation of the meetings of the International Dental Federation in London and Cambridge. On the contrary, the members that have worked on this question should be thanked by the Council.

Drs. Godon and Sauvez said that the Council had worked regularly on the organization of these meetings, and that it was necessary, considering that the meetings were going to be held in London, to profit by the presence of Dr. Cunningham in Paris for the preparatory meetings. They accepted the amendment proposed by Dr. Aguilar, and said that they thought it was possible to satisfy him by admitting that during the year there had been held two meetings of the Subcommittee for the preparation of the by-laws, and three meetings of the Council, in which Dr. Cunningham took part to help the Council in the organization of the London meetings.

With these modifications, the minutes were approved.

The secretary-general then read his report on the organization of the Commission of Education.

This report was adopted.

On account of the lateness of the hour, the remaining items in the order of business were deferred to the next meeting.

Adjourned at ten P.M.

*London, August 5, 1900.*

The meeting was called to order by President Godon at four P.M.

The following members were present: Drs. Aguilar, Cunningham, Förberg, Harlan, Hesse, and Sauvez.

Dr. Sauvez, the secretary-general, read the minutes of the meeting of the preceding day. He announced the order of business, and read the following letters:

1. A letter from Drs. Förberg, Forssman, Sandstedt, and Christensen, proposing that the next meeting of the Executive Council of the International Dental Federation be held in Stockholm.

The discussion of this proposition was deferred to the meeting of August 7, in Cambridge.

2. A letter of Professor Limberg, excusing himself for his inability to be present at the meetings of 1901, expressing regret at the misunderstandings of the closing session of the Congress, and announcing that a congress of Russian dentists, to be held in Odessa in 1902, will decide whether a member shall be appointed by the Russian dentists to the Commission of Education.

3. A letter of Dr. Rosenthal, presenting his resignation, which was unanimously rejected by the Council.

Dr. Sauvez then read his report, which here follows:

GENTLEMEN AND HONORED CONFRERES,—Permit me to thank you for the great honor that you have conferred upon me by appointing me secretary of the Executive Council of the International Dental Federation.

The second meeting of the Council will take place to-day, beginning the labors which will come to an end in Cambridge, August 7.

The report that I read to you at the beginning of this meeting has informed you of the present situation.

You will recall the creation of the International Dental Federation. It was at the last meeting of the International Dental Congress in Paris, 1900, that it was decided to organize this Federation, in accordance with the requests presented to and accepted by the Congress. You will also remember that at that meeting we were appointed members of the Executive Council. I mention these points simply because I want you to remember that we are the legal representatives of the twelve hundred members of the International Dental Congress. Our powers have been transmitted to us by that body, and will expire at the next Congress. We have emanated directly from the Congress and we must report at its next meeting the course and result of our work.

The purpose of the Federation, as manifested in its constitution, consists especially in providing for international dental congresses and reunions; in maintaining and in extending the relations between the several national committees and societies, and in organizing the several international commissions that it may deem necessary to create; in *résumé*, the International Dental Federation must organize everything that may contribute to the advancement of odontological science.

If that be the purpose of the Federation, then the *rôle* of its Executive Council must be to plan the regulations of the Federation, to supervise their execution, to fix the dates and places of meeting of the international reunions, to summon the several commissions, to carry out the decisions of the Congress, and to examine the propositions submitted to it.

It is according to this order of ideas and in this spirit that we have been working since our last meeting. You will remember that a subcommittee of three members was appointed in order to insure the preparation of the constitution during the year 1900-1901. That committee held its first meeting on November 28, 1900, in Paris, at Dr. Godon's residence. The Subcommittee decided among other things upon the publication of the *Bulletin of the Executive Council of the International Dental Federation* in four languages; this bulletin to serve as the official organ of the Executive Council. *L'Odontologie* has undertaken gratuitously the publication, and several foreign journals have courteously republished the text of said bulletin. We tender them our thanks in the name of the Council.

The members of the Executive Council and of the International Commission of Education have been notified of their appointment to said committees.

A second meeting of the subcommittee was held in Paris, May 27, also at Dr. Godon's residence, as reported in the proceedings. It was at that meeting that the Subcommittee prepared the regulations that we will presently submit to your consideration. It was also at that meeting that we organized the present session of the Council in London, profiting by the attraction offered by the annual meeting of the British Dental Association and of the American Dental Society of Europe.

We have reason to feel satisfied with the result obtained, for all the members that accepted the nominations agreed also to come here. Dr. Pichler is the only one that resigned, and his resignation was on account of ill health.

This first meeting is starting under happy auspices, and is being made delightful by the most cordial reception and the kind and generous hospitality tendered us by our English *confrères.* I think that I shall be acting as your interpreter in asking Mr. Cunningham to transmit our sincere thanks to the president of the British Dental Association.

This first session will emphasize in an indisputable manner the existence of the Executive Council, and hence of the International Dental Federation. This first session seems to assure us that the coming meetings will be followed with as much attention even by the gentlemen of far-off countries, such as Dr. Harlan, and that the preparation for the next Congress, which is our principal *raison d'être,* will be made with the assured assistance of the *confrères* best qualified for this kind of work. It is not our intention in these too short sessions to especially call your attention to the past; we will direct our efforts to the present and future. Consequently we must show you how important it is that the national federations and the various societies should cultivate intimate relations with the Executive Council,—not because the Council desires to interfere in questions of a national character. The Council acts only as a counsellor and organizer, leaving every federation or society completely free and independent, and master of its own acts each in its respective country. This Council must form the legal union between the societies of different nations.

We must also endeavor to represent this Council in our various countries not as though it were a sort of secret committee occupied with great international questions, but as a committee of union, solidarity, and confraternity, ready to receive all the suggestions, all the propositions of the professional federations or societies, and to study them in order to reach impartial conclusions. We must represent that Council conscious of the fact that it is the outcome of a universal election, and that it is a body beneficial, if not essential, to the good understanding and confraternity of the dentists of all countries. Our particular personalities cease to exist; they will modify themselves and disappear. The body alone exists; the cells which compose it are called upon to undergo transformations, to grow old, to be replaced by others; but that matters not. What we must insure is the existence, the vitality, the power of the organ, which is the Executive Council, the active part and the representative of the International Dental Federation. This is why all the efforts of the members must tend to insure the good relations of the Council with the federations.

Our efforts should also be directed towards the formation of solid and well-united federations between the national societies. In France all our efforts have been made towards that end, and we can announce to-day that the French Dental Federation is solidly established. Its first session will be held in Corsica in September, as the Section on Odontology of the French Association for the Advancement of Science, thanks to the efforts of President Godon.

I have yet to speak to you on the Commission of Education, on its organization, and on the work we are going to ask them to perform.

These several points will be the objects of a new report, which will be presented to you in the near future.

In the mean while I beg you, gentlemen and honored *confrères*, to pardon my having occupied your attention for such a long time. Before concluding, I will also ask you to thank Mr. Cunningham, the principal organizer of this session, who has been instrumental in assuring us the honor of a reception in Cambridge, in that ancient university whose reputation has been world-wide for centuries.

The Council adopted this report, and adjourned until August 6.

*London, August 6, 1901.—Morning Session.*

The meeting was called to order at nine A.M. by President Godon.

The following members were present: Drs. Aguilar, Cunningham, Frank, Harlan, Hesse, and Sauvez.

Dr. Sauvez, the secretary-general, read the minutes of the previous session, which were approved.

The secretary-general proposed the names of Drs. Bryan, Zsigmondy, and Haderup for appointment to the Commission of Education.

The next thing in order should have been the election of officers, but Dr. Frank suggested that before doing this the question of the regulations of the International Dental Federation be taken up.

The meeting then adjourned, after having decided to hold the next meeting on the same day at two P.M.

*Afternoon Session.*

The meeting was called to order at two P.M.

The following members were present: Drs. Aguilar, Cunningham, Förberg, Frank, Harlan, Hesse, and Sauvez.

The secretary-general read the minutes of the previous session, which were adopted.

The Council decided that at its last meeting a vote of thanks should be addressed to the Royal Temple Yacht Club.

The president asked the Council to place the election of officers at the head of the order of business. He thought that, considering the international character of the Council, a new president should be elected, and refused to remain chairman.

Dr. Sauvez made a few remarks of a similar nature to those of Dr. Godon.

The Council then proceeded to vote for the appointment of officers. The result of the vote for president was as follows: Dr. Godon, 5; Dr. Cunningham, 2.

Dr. Godon declined to accept the chairmanship, and asked for a second balloting, which, however, gave the same results.

The balloting for vice-presidents gave the following results: Dr. Cunningham, 6; Dr. Förberg, 4; Dr. Harlan, 1; Dr. Hesse, 1. Consequently Drs. Cunningham and Förberg were elected vice-presidents.

The Council decided not to vote for secretary-general, but to appoint Dr. Sauvez by acclamation.

On motion of Dr. Brophy, Dr. Pearson, of Canada, was appointed adjunct member of the Commission of Education.

The meeting adjourned at three P.M.

*Cambridge, August 7, 1901.*

This meeting took place at Trinity College Hall, Cambridge, at six P.M.

The following members were present: Drs. Aguilar, Cunningham, Frank, Harlan, Hesse, and Grevers.

Dr. Sauvez read the minutes of the previous session.

The only question that was discussed was that of fixing the next place of meeting of the Executive Council. The members present were of the opinion that the meeting should be at Stockholm, as proposed by Dr. Förberg and his colleagues.

The meeting then adjourned.

*Cambridge, August 8, 1901.*

The Council was called to order at nine P.M. by President Godon.

Dr. Sauvez read the minutes of the previous meeting, which were approved.

The plan of appointing a Commission of Public Dental Hygiene was adopted, and a discussion as to the manner in which this commission should be appointed then followed, in which Drs. Sauvez, Aguilar, and Cunningham took part.

A commission of three members was then appointed for one year, this commission to present a report at the next meeting of the Executive Council. Drs. Förberg, Frank, and Cunningham were appointed to this commission. Dr. Frank was intrusted with the initial work of the Commission of Public Dental Hygiene.

Dr. Aguilar asked that it should be plainly specified that the Executive Council represents the several countries. He made a motion, which was amended by Dr. Godon, and which reads as follows:

The International Dental Federation is represented by an Executive Council composed of (1) Members elected by the Congress which represented the different countries.

At this point Dr. Brophy was introduced. He read a telegram from the National Association of Dental Faculties, in which information was given of the extension of the course in dentistry in the United States to four years.

The president requested Dr. Brophy to convey to the Association the thanks and congratulations of the International Dental Federation.

The Council then discussed the question of appointing adjunct members. The question was voted upon and adopted.

Dr. Sauvez presented an amendment, which was also adopted. (This motion, with its amendment, corresponds to Article IX. of the regulations of the International Dental Federation.)

Dr. Sauvez then presented another amendment to Article XIV. of the regulations; this, however, was rejected. The amendment referred to the power of the Council, following the recommendation of the national federations, to appoint members to the Commission of Education.

The following article was added to the regulations:

Article XV.—These regulations are adopted for one year, and shall be revised at the next session of the Federation.

The question of deciding upon the date and place of meeting of the next International Dental Congress was referred to the next meeting of the Federation, to be held in Stockholm.

It was decided to invite the Russian National Committee to send an adjunct member to the Executive Council and an active member for the Commission of Education.

On motion of Dr. Frank, the Austrian National Committee will be invited to appoint a member to the Executive Council as a substitute for Dr. Pichler, who resigned.

The Council congratulated the National Austrian Committee on having sent Dr. Frank to the first session.

It was decided to send the thanks of the Council to the Royal

Temple Yacht Club, to the British Dental Association, and to Dr. Cunningham for their contributions towards the success of the meetings.

The members of the Council were commissioned to prepare reports, to be sent to the secretary-general, concerning the condition of dental affairs in every country, and the number and addresses of the schools, of societies, of journals, and of dentists.

The officers of the Council were invited to present at Stockholm a report concerning the changes to be made in the Council with regard to the number of members of which it should be composed.

It was also decided that each national dental federation should confirm the powers of its delegates to appoint representatives.

Adjourned at eleven A.M.—*Dental Cosmos.*

(To be Continued.)

